# Truncating mutations in the Wilson disease gene ATP7B are associated with very low serum ceruloplasmin oxidase activity and an early onset of Wilson disease

**DOI:** 10.1186/1471-230X-10-8

**Published:** 2010-01-18

**Authors:** Uta Merle, Karl Heinz Weiss, Christoph Eisenbach, Sabine Tuma, Peter Ferenci, Wolfgang Stremmel

**Affiliations:** 1 Department of Gastroenterology and Hepatology, University Hospital, Heidelberg, Germany; 2 Department of Gastroenterology and Hepatology, Medical University of Vienna, Vienna, Austria

## Abstract

**Background:**

Mutations in the gene ATP7B cause Wilson disease, a copper storage disorder with a high phenotypic and genetic heterogeneity. We aimed to evaluate whether 'severe' protein-truncating ATP7B mutations (SMs) are associated with low serum ceruloplasmin oxidase activities and an early age of onset when compared to missense mutations (MMs).

**Methods:**

The clinical phenotype of 59 genetically confirmed WD patients was analyzed retrospectively. Serum ceruloplasmin was measured by its oxidase activity with *o*-dianisidine dihydrochloride as substrate and immunologically.

**Results:**

Thirty-nine patients had two MMs, 15 had the genotype SM/MM, and 5 patients had two SMs on their ATP7B alleles. Enzymatic and immunologic serum ceruloplasmin levels differed significantly between the three groups (P < 0.001 and P < 0.01, respectively). The lowest levels were measured in patients with two SMs (0.0 U/L; IQR, 0.0-0.0 U/L and 0.02 g/L; IQR, 0.01-0.02 g/L, respectively) and the highest in patients with two MMs (17.8 U/L; IQR, 5.8-35.1 U/L and 0.11 g/L; IQR,0.10-0.17 g/L, respectively). The age of onset was also significantly different between the three patient groups (P < 0.05), with SM/SM patients showing the earliest onset (13 years; IQR, 9-13 years) and patients with two MMs showing the latest onset (22 years; IQR, 14-27 years). By ROC curve analysis a ceruloplasmin oxidase level ≤ 5 U/L can predict the presence of at least one SM with a sensitivity of 80% and a specificity of 79.5%.

**Conclusions:**

In our German study cohort truncating ATP7B mutations were associated with lower ceruloplasmin serum oxidase levels and an earlier age of onset when compared to MMs. Measurement of serum ceruloplasmin oxidase might help to predict presence of truncating ATP7B mutations and might facilitate the mutation analysis.

## Background

Wilson disease (WD) is a rare, autosomal-recessively inherited disorder of copper metabolism due to mutations of the WD gene ATP7B [1,2]. The WD gene ATP7B codes for a membrane-bound, copper-binding protein that is expressed primarily in the liver [1,2]. Disease causing mutations in the ATP7B gene result in a reduced biliary copper excretion [3]. WD is clinically characterized by hepatic (e.g. liver cirrhosis) and neurological manifestations related to the accumulation of copper in the liver and the brain [3]. The onset of signs and symptoms of WD typically is in childhood and young adulthood but may even be much later [4].

In addition to copper accumulation the disturbed function of ATP7B results in an impaired incorporation of copper into apoceruloplasmin leading to the formation of an unstable ceruloplasmin which is rapidly degraded [5]. Although this might be of minor importance in copper homeostasis, impaired holo-ceruloplasmin synthesis is of diagnostic relevance as serum ceruloplasmin concentration is considered a useful laboratory test for diagnosis of WD [6]. Serum ceruloplasmin concentration is determined in most routine laboratories immunologically, but can also be measured enzymatically by its oxidase activity [7-9]. Recently, we could show that measurement of the enzymatic oxidase activity of serum ceruloplasmin with o-dianisidine dihydrochloride as substrate is of greater diagnostic value than its immunologically measured concentration [10].

WD typically begins with a pre-symptomatic period, during which copper accumulation in the liver leads to subclinical hepatitis, and progresses to liver cirrhosis and development of neuropsychiatric symptoms. The type of hepatic and neurological symptoms can be highly variable. The wide variation in the clinical phenotype of WD has still to be elucidated and may be related to the ATP7B genotype. Although some authors have tried to establish whether the ATP7B genotype determines the phenotype of the disease, the data are conflicting and no definitive association has been established [11-14]. Reasons for the difficulties in assessing genotype-phenotype correlations in WD are the high genetic heterogeneity with the large number of mutations and the rareness of the disease. About 300 mutations have been described (Wilson Disease Mutation Database compiled by the Department of Medical Genetics at the University of Alberta is accessible at the web page http://www.wilsondisease.med.ualberta.ca/database.asp). Protein-truncating nonsense mutations and frameshift mutations are presumed to have a different impact on the functional impairment of the ATP7B protein than missense mutations and are hypothetically more 'severe' mutations than these. In line with this assumption, a more severe impairment of copper metabolism parameters and an earlier age of disease onset in WD patients with protein-truncating mutations could recently be demonstrated for different European cohorts [15,16].

The aim of our study conducted in a German WD cohort was to evaluate whether the type of mutation correlates (1) with serum ceruloplasmin levels measured enzymatically with o-dianisidine as substrate, and (2) with age of onset of the disease.

## Methods

### Patients

One hundred and ten consecutive patients with WD (45 men and 65 women), all referred to the Department of Gastroenterology at the University of Heidelberg between 04/2006 and 04/2008, were screened. In all screened patients diagnosis of WD was based on the WD score published previously [17] and mutation analysis was performed as described previously [4]. In 62 of these screened patients mutation analysis revealed a homozygous or compound heterozygous ATP7B mutation. Exclusion criteria were an acute hepatitis (n = 0), pregnancy (n = 0), and intake of anticonceptive pills (n = 3) as ceruloplasmin may be elevated in inflammatory stages, during pregnancy, and in response to exogenous administration of estrogens [18,19]. After exclusion of all women taking anticonceptive pills the remaining 59 genetically-confirmed subjects (25 men, 34 women) were enrolled into the study. Of these 59 patients, 4 were treatment naïve and 55 were treated with D-penicillamine, trientine, or zinc salts.

The study protocol was approved by the ethics committee of the University of Heidelberg and all patients gave written informed consent.

### Laboratory methods

Blood was collected after an overnight fast. Sera were collected and stored (for 4 to 28 months) at -80°C until assayed. Total serum ceruloplasmin concentration was analyzed by the Department of clinical chemistry of the University hospital Heidelberg using an immuno-nephelometric assay with anti-serum to human ceruloplasmin (Dade Behring, Marburg, Germany) on a BNII nephelometer (Dade Behring, Germany) according to the manufacturer's instructions.

Serum ceruloplasmin oxidase activity with *o*-dianisidine dihydrochloride as substrate was determined spectrophotometrically as described previously [10].

### Statistical analysis

Variables are expressed as median with interquartile range (IQR). The data obtained for ceruloplasmin oxidase activity, immunoreactive ceruloplasmin concentration, and age are summarized as box and whisker plots. For statistical analysis of differences between the groups the Kruskal-Wallis-test with *post-hoc *testing using Mann-Whitney U-test was used. 'Initial presentation' was compared between groups by Fisher's exact test. McNemar test was used to compare sensitivity and specificity of both ceruloplasmin assays for prediction of presence of SMs. All calculations including generation of receiver operating characteristics (ROC) analysis were performed using SPSS version 16.0 (SPSS, Chicago, IL). Two sided p-values were reported in all cases and a p-value < 0.05 was considered statistically significant.

## Results

### Analysis of mutations of ATP7B

The most common mutation was the missense p.H1069Q mutation, being homozygous in 30 (50.9%) and compound heterozygous in 15 (25.4%) patients. Other infrequent missense mutations were p.R710Q (detected in n = 1 patients), pE754K (n = 1), p.P760L (n = 1), p.D765N (n = 3), p.M769V (n = 1), p.R778L (n = 1), p.T858A (n = 1), p.R919W (n = 1), p.R969Q (n = 2), p.G982V (n = 1), p.A1063V (n = 1), p.G1099S (n = 1), p.V1106D (n = 1). Detected nonsense mutations were p.Y741X (n = 1), p.W779X (n = 4), and p.L1088X (n = 3). The frameshift mutations detected were p.L722E-fs (n = 1), p.P767P-fs (n = 2), c.778dupC (n = 1), p.K844K-fs (n = 2), c.del930-935 (n = 1), p.Val1217_Leu1218del (n = 2), p.I1330I-fs (n = 1), c.3402delC (n = 2), and c.3843insT (n = 1). One splice site mutation was found (c.1708-1G>A, n = 1)).

Of all 27 identified mutations 4 were novel. The novel mutations included c.del930-935 (n = 1), p.I1330I-fs (n = 1), c.3843insT (n = 1), and pE754K (n = 1).

Most mutations were detected in exons 8 (10 different mutations) and 15 (4 different mutations). No mutation was detected by means of direct sequencing in exons 3 to 7, 9, 11, 16, and 20. Of the 27 detected mutations in ATP7B, 15 were classified as mild mutations ('MMs', missense mutations), and 12 as severe mutations ('SMs', frameshift/nonsense/splice site mutations).

### Evaluation of the correlation of phenotype and genotype

Of the 59 patients analyzed, 39 (66.1%) had two MMs (MM/MM), 15 (25.4%) had the genotype SM/MM, and 5 patients (8.5%) had two SMs on their ATP7B alleles (SM/SM).

There were no statistically significant differences between the types of mutation and the mode of initial presentation: Of the 39 WD patients carrying only missense mutations primary clinical symptoms were neurological in 7 and hepatic in 32. Of the 15 patients with an SM/MM genotype primary clinical symptoms were neurological in 5 and hepatic in 10. In the five patients with two SMs primary symptoms were neurological in 2 and hepatic in 3.

**Figure 1:**
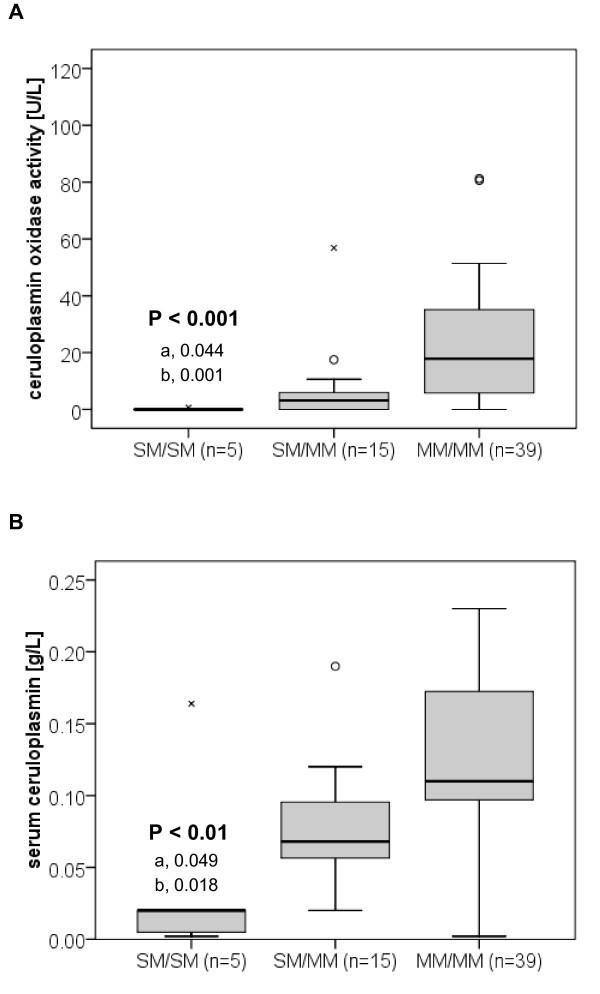
**Serum ceruloplasmin oxidase activity and immunoreactive serum ceruloplasmin in Wilson disease patients grouped according to their type of ATP7B mutation**. Analysis of Wilson disease (WD) patients possessing either (1) two 'severe' mutations (SM/SM); (2) one 'severe' mutation and one missense mutation (SM/MM), or (3) two missense mutations (MM/MM). Distribution of data presented as box and whisker plots: 25^th ^percentile, median, 75^th ^percentile, maximum, outliers (circles), and extreme values (x). Both assays resulted in no indeterminate results or missing data. a, p-value for the comparison SM/SM with SM/MM patients; b, p-value for the comparison SM/SM with MM/MM patients. (A) Oxidase activity is significantly lower in SM/SM patients (0.0 U/L; IQR, 0.0 to 0.0) compared to SM/MM patients (3.1 U/L; IQR, 0.0 - 5.9) and MM/MM patients (17.8 U/L; IQR, 5.8 - 35.1). (B) Immunoreactive serum ceruloplasmin is significantly lower in SM/SM patients (0.02 g/L; IQR, 0.01 - 0.02) compared to SM/MM patients (0.07, g/L; IQR 0.06 - 0.10) and MM/MM patients (0.11 g/L; IQR, 0.10 - 0.17).

Serum ceruloplasmin levels differed significantly between the three groups. This was true for both enzymatically and immunologically measured serum ceruloplasmin levels (P < 0.001 and P = 0.001, respectively). The lowest levels were measured in patients with two SMs (0.0 U/L; IQR, 0.0 - 0.0 U/L and 0.02 g/L; IQR, 0.01-0.02 g/L, respectively) and the highest in patients with two MMs (17.8 U/L; IQR, 5.8 - 35.1 U/L and 0.11 g/L; IQR, 0.10 - 0.17 g/L, respectively) (Figure 1A and 1B).

**Figure 2:**
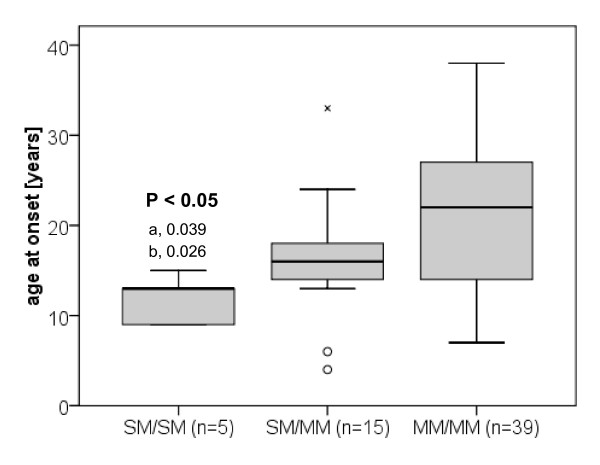
**Age of disease onset in Wilson disease patients grouped according to their type of ATP7B mutation**. Analysis of Wilson disease (WD) patients possessing either (1) two 'severe' mutations (SM/SM); (2) one 'severe' mutation and one missense mutation (SM/MM), or (3) two missense mutations (MM/MM). Distribution of data presented as box and whisker plots: 25^th ^percentile, median, 75^th ^percentile, maximum, outliers (circles), and extreme values (x). Both assays resulted in no indeterminate results or missing data. a, difference for the comparison with SM/MM patients; b, difference for the comparison with MM/MM patients. Age of onset is significantly earlier in SM/SM patients (13 years; IQR, 9 - 13) compared to SM/MM patients (16 years; IQR, 14 - 18) and MM/MM patients (22 years; IQR, 14 - 27).

The age of disease onset was also significantly different between the three patient groups (P = 0.019). Patients with two SMs manifested their disease at the lowest age (13 years; IQR 9-13), while patients with two MMs were oldest at disease onset (22 years; IQR 14 to 27) (Figure 2).

**Figure 3:**
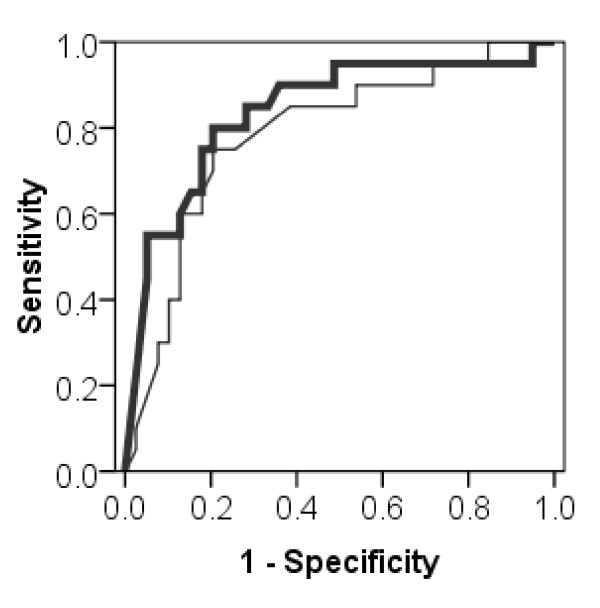
**ROC (receiver operating characteristic) curves for the prediction of presence of 'severe' ATP7B mutations by serum ceruloplasmin oxidase activity and immunoreactive serum ceruloplasmin**. For serum ceruloplasmin oxidase activity (bold line) the area under the curve is 0.838 (95% CI, 0.724 - 0.953) and for immunoreactive ceruloplasmin (light line) the area under the curve is 0.785 (95% CI, 0.658 - 0.911).

By ROC curve we analyzed if ceruloplasmin serum levels predict the presence of SMs. For ROC curve analysis patients were grouped into two groups: patients with at least one SM (n = 20) and patients with a homozygous or compound heterozygous MM (n = 39). ROC curve analysis resulted in the greatest sum of sensitivity and specificity of serum ceruloplasmin oxidase activity in identifying WD patients with at least one SM at a cut point of 5 U/L, while the greatest sum of sensitivity and specificity of immunoreactive ceruloplasmin concentrations in our study cohort was obtained at a cut point of 0.09 g/L (Figure 3). For ceruloplasmin oxidase activity the sensitivity was 80.0% (95% CI, 55.7% - 93.4%) and the specificity was 79.5% (95% CI, 63.1% - 90.1%) at a cut-off of ≤ 5 U/L. For immunoreactive ceruloplasmin the sensitivity was 75.0% (95% CI, 50.6% - 90.4%), and the specificity was 79.5% (95% CI, 63.1% - 90.1%) at a cut-off of ≤ 0.09 g/L. The ROC curve analysis showed that both, serum ceruloplasmin oxidase activity and immunoreactive serum ceruloplasmin, can be used to predict the presence of a SM. McNemar test revealed no significant difference between both assays in predicting presence of SMs, neither for sensitivity nor for specificity.

## Discussion

Although considered a useful diagnostic test in WD, immunologically measured serum ceruloplasmin has been shown to be in the normal range in 35% of patients with hepatic WD [20] and a low immunologically measured serum ceruloplasmin has a positive predictive value of only 5.9% [21]. As apoceruloplasmin may interfere with the immunologic assay but not with the enzymatic ceruloplasmin measurement, an overestimation of serum ceruloplasmin can be avoided by use of the oxidase method for ceruloplasmin determination. Recently we could provide evidence that serum ceruloplasmin oxidase activity measured enzymatically with o-dianisidine as substrate is superior to immunoreactive ceruloplasmin in diagnosing WD.

In our present study we analyzed if serum ceruloplasmin oxidase levels correlate with the ATP7B genotype. The patients were grouped according to the mutations detected in protein truncating and missense mutations. This grouping was based on the hypothesis that truncating and non-truncating mutation groups are likely to have a different impact in the functional properties of the mutated protein. As serum ceruloplasmin levels can increase in response to pregnancy, estrogen therapy, or acute inflammation, patients fulfilling these criteria were excluded from the study cohort [18,19].

For the first time we could show that serum ceruloplasmin oxidase activities measured enzymatically with o-dianisidine as substrate differ significantly between WD patients with protein-truncating and non-truncating ATP7B mutations. In our German WD cohort of 59 patients protein-truncating mutations were associated with significantly lower oxidase levels than missense mutations. In addition to ceruloplasmin oxidase activities we determined immunoreactive ceruloplasmin serum levels and could show the same relation. Our observation is in line with previously published studies that were conducted in other European study cohorts: one Greek, one Polish, and one Italian [15,16,22]. In these studies patients with protein-truncating mutations had lower ceruloplasmin concentrations than patients with missense mutations. This is of note, because it augments the line of evidence for an association of protein-truncating mutations with a more severe impairment of holo-ceruloplasmin synthesis. It might explain normal or borderline ceruloplasmin levels in some WD patients as protein-truncating mutations disrupt any copper-transporting function, while certain missense mutations might allow residual function in holo-ceruloplasmin synthesis.

In parallel to the effect on serum ceruloplasmin oxidase activities we observed an earlier onset of WD in patients with truncating ATP7B mutations when compared to missense mutations. This finding in our German study cohort is in accordance with two previously published studies conducted in European cohorts, a Polish and a Greek [15,22]. However, other reports did not find a younger age of onset in patients with truncating mutations [16,23,24]. The effect of truncating mutations observed in our present study might be influenced by the p.H1069Q mutation, the most frequent mutation in European WD patients. For this mutation an association with a late and predominantly neurological presentation in WD could be shown in a recent meta-analysis [11], although the underlying studies are quite controversial [12-14]. Thus the predominance of the homozygous pH1069Q mutation in the MMs group in our study cohort might have biased the 'group effect' seen for MMs when compared to truncating mutations. Nevertheless, our results support the line of evidence that at least for European WD patients an influence of the type of mutation on the phenotype can be assumed.

Because ATP7B mutation analysis, due to the great heterogeneity of mutations, is quite laborious, we asked if serum ceruloplasmin levels might allow prediction of truncating mutations. By ROC analysis ceruloplasmin level cut-points with the highest sum of sensitivity and specificity for prediction of truncating mutations were identified. At an optimal cut-point ceruloplasmin oxidase activity allowed prediction of SMs with a sensitivity of 80% and a specificity of 79.5%. Based on this result, mutation analysis in European WD patients might be facilitated by considering serum ceruloplasmin oxidase level before starting mutation analysis, as assuming the more probable type of mutation before starting mutation analysis might shorten the mutation identification process. However, as discussed above, the results of ROC analysis might only apply for European patients and not for populations with a different mutation spectrum.

## Conclusion

In conclusion, our data show that in our German study cohort truncating ATP7B mutations were associated with lower serum ceruloplasmin oxidase levels and an earlier age of disease onset when compared to MMs. At least in European populations measurement of serum ceruloplasmin oxidase might help to predict presence of truncating ATP7B mutations and might facilitate the mutation analysis.

## Abbreviations

WD: Wilson disease; ROC: receiver operating characteristics; CI: confidence interval.

## Competing interests

The authors declare that they have no competing interests.

## Authors' contributions

UM conceived of the study and its design, collected the data, performed the statistical analysis and drafted the manuscript. KHW participated in data acquisition and helped to draft the manuscript. CE helped in interpretation of data and to draft the manuscript. ST carried out the ceruloplasmin oxidase activity measurements and participated in data acquisition. PF carried out mutation analysis and helped to draft the manuscript. WS helped to draft the manuscript. All authors read and approved the final manuscript.

## Pre-publication history

The pre-publication history for this paper can be accessed here:

http://www.biomedcentral.com/1471-230X/10/8/prepub
